# Anti-coagulation therapy following coronary endarterectomy in patient with coronary artery bypass graft

**DOI:** 10.22088/cjim.9.1.27

**Published:** 2018

**Authors:** Hamdi Reza Vafaey, Mohammad Taghi Salehi Omran, Sadaf Abbaspour, Nadia Banihashem, Ghassem Faghanzadeh Ganji

**Affiliations:** 1 Department of Cardiac Surgery, Ayatollah Rouhani Hospital, Babol University of Medical Sciences, Babol, Iran; 2 Department of Cardiology, Ayatollah Rouhani hospital, Babol University of Medical Sciences, Babol, Iran; 3 Student Research Committee, Babol University of Medical Sciences, Babol, Iran; 4 Department of Anesthesiology, Babol University of Medical Sciences, Babol, Iran

**Keywords:** Coronary artery bypass graft, Coronary endarterectomy, Warfarin, Clopidogrel

## Abstract

**Background:**

Since there is a lack of research on postoperative anticoagulation protocol in patients undergoing coronary artery bypass graft (CABG) / coronary endarterectomy (CE), we recommend a new protocol for anticoagulation in these patients.

**Methods:**

In this double-blind randomized clinical trial study, 52 patients undergoing CABG / CE entered the study and were divided into two groups. In group 1, the patients were given warfarin(international normalized ratio (INR) between 2-3) together with 80 mg aspirin daily for 3 months. In group 2, the patients were given 75 mg plavix daily together with 80 mg aspirin daily for 3 months. We evaluated patients with electrocardiography, echocardiography and checking ceratin phosphokinase MB and troponin I in the several stages. The data were analysed SPSS Version18 software.

**Results:**

There was no significant difference between pre and post-operative Ejection fraction in patients with plavix (P=0.21) and warfarin (P=0.316) regimen. However, wall mrotion score was significantly better in clopidogrel – aspirin patients in late (3 months) post operation (p<0.001).

**Conclusions:**

Since warfarin has serious hemorrhagic complications and requires closed monitoring of serum drug activity by serial INR checking, it is recommended that clopidogrel – aspirin can be the preferred alternative anticoagulation therapy in CABG / CE patients.

Full myocardial revascularization is the essential primary aim in the treatment of coronary artery disease (CAD) and is the most important factor that recognizes surgical revascularization from other forms of revascularization such as percutaneous coronary intervention (PCI) (1-3). Before presentation of coronary artery bypass grafting (CABG) as the gold standard in the surgical treatment of CAD, coronary endarterectomy (CE) was one of surgical techniques recommended for the management of complex coronary artery stenoses (4). Theoretically, combination of these two techniques (CABG/CE) can lead to more complete myocardial revascularization in complex CADs. This is especially important in patients with diffuse CAD, because they usually have limited available target vessels for CABG (5, 6). There are numerous reports that in early postoperative period, CABG/ CE in comparison with isolated CABG puts the patients in poorer outcomes in 30-days follow up (7, 8). Many efforts have been made to maintain patency of endarterectomized coronary grafts in postoperative period and to prevent such graft failure and subsequent perioperative myocardial infarction (MI) in that territory. Generally, as routine practice in patients with CABG / CE, it is recommended that warfarin in combination with aspirin should be continued until 3 months postoperatively and eventually aspirin alone permanently. 

Warfarin therapy has some serious potential side effects such as bleeding, tissue necrosis and hypersensitivity reactions .Thus ,patients must be evaluated weekly for International normalized ratio (INR) testing to keep it around 2-3. On the other hand, several studies documented the combination of aspirin – clopidogrel as an effective treatment in acute MI (8, 9). So the combination of aspirin – clopidogrel has been recognized as gold standard treatment in acute coronary syndromes aiming inhibition of platelet aggregation. One of the benefits of clopidogrel is that there is no need to test INR once a week. Since there is a lack of research on postoperative anticoagulation protocol in patients with CABG / CE, we decided to conduct this study as a clinical trial to compare the outcomes in two such protocols: clopidogrel – aspirin and warfarin – aspirin.

## Methods

This study was a randomized, double-blind, clinical trial performed at Ayatollah Rouhani Hospital, Babol, Iran. A total of 55 consecutive patients undergoing CABG / CE with cardiopulmonary bypass (CPB) from May 2012 to June 2014 were evaluated. All patients gave an informed consent to enter the study. This study was approved by the Ethics Committee of Babol University of Medical Sciences (IRCT2016102730410N2). Exclusion criteria were as follows: a need to concomitant valve surgery, history of allergic reaction to medications used in the study and any comorbidity (renal and hepatic failure, respiratory insufficiency). 

All patients underwent standard CABG / CE with CPB and the same technique of intermittent antegrade cold blood cardioplegia. In all cases, decision to endarterectomy was made if the lumen of coronary artery was occluded totally, seemingly that acceptable grafting would be impossible without endarterectomy. To perform an endarterectomy, target coronary artery was incised longitudinally 1.5 to 2.5 centimeters along with atherosclerotic plaque which would be removed and then after “closed “endarterectomy by aid of a dissector, the plaque was excised with secure distal tapering. In postoperative period, the patients were divided into two groups randomly. In group “warfarin – aspirin “as routine practice in literature, from the first postoperative day, aspirin was taken by the patients, in addition to warfarin to reach target range of INR 2-3 days. In “clopidogrel -aspirin “group, from first postoperative day, 80 mg aspirin was given to thepatients plus 75 mg clopidogrel. All data of both groups were collected and analyzed by a blinded cardiologist. These data included ECG (new Q wave and new ST-T changes) and cardiac enzymes (cardiac troponin I and CPK”MB”). These parameters serially checked intervals including just pre-and-post operation, after 24 hours and 48 hours post operation. An echocardiography was also performed in all patients in preparation and 3 month post operation evaluating ejection fraction (EF) and wall motion abnormality. Wall motion abnormality was quantified by wall motion score index (WMSI). All 16 cardiac segments were evaluated in WMSI.Wall motion score will be 1 if wall motion is normal and it will be 2 if wall motion is hypokinetic. Wall motion score in akinetic wall or scar tissue was 3. The mean value of scores of all 16 cardiac segments is known as “total”WMSI but “local”WMSI is the mean value of scores of segments of a special coronary artery territory. The statistical analysis software (Version 18, SPSS) was used for all analyses. All data were analysed using the chi – square test, Fisher‘s exact test and t- test. A p-value less than 0.05 was considered significant.

## Results

**Figure1:**
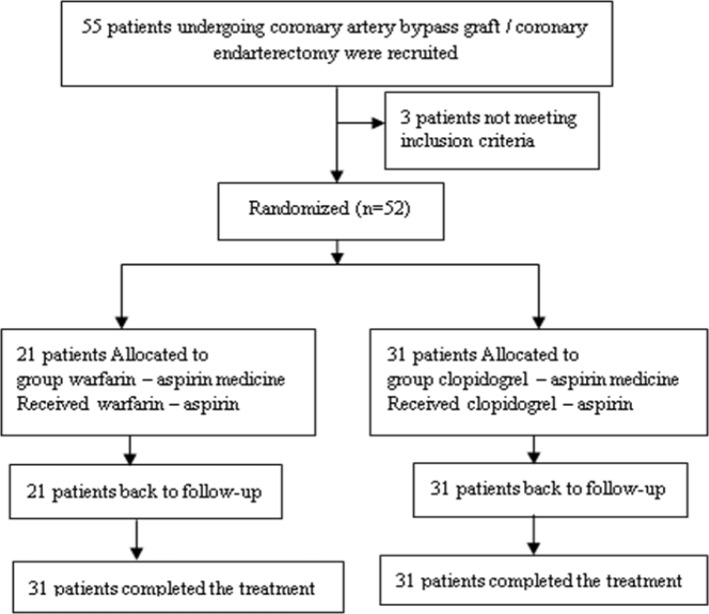
Schematic representation of groups in study

From 55 patients with CABG / CE, 3 patients were excluded. 52 remaining patients were divided into two groups randomly. 31 patients were in clopidogrel – aspirin group (including 19 males and 12 females) while 21 patients were in warfarin – aspirin group (including 10 males and 11 females) (figure1).

The mean age in clopidogrel – aspirin group was 62.9±9.85 years and in warfarin – aspirin group was 58.86±11.42 years. This was not statistically significant. According to sex, there was no statistically significant difference between two groups. From all cases of endarterectomy, 19 (36.5%) cases were in left anterior descending (LAD) coronary artery (including 7 ones in warfarin group and 12 ones in clopidogrel group) and 33 (63.5%) cases in the right coronary artery (RCA) (including 14 cases in warfarin group and 19 cases in clopidogrel group). The mean ICU stay was 4.6±1.9 days in clopidogrel group and 4.63±1.29 days in warfarin group. This difference was not statistically significant (P=0.949). The mean hospital stay was 9.07±3.29 days in clopidogrel group and 9.26±1.93 days in warfarin group (p=0.820). ECG was obtained immediately, 24h and 48h postoperation showed no new Q wave nor new ST-T changes in two groups of patients. 

Serum CPK(MB) level was checked for any association before surgery and immediately after surgery, in addition to 24 and 48h later. With the attention to increase postoperative serum CPK (MB) level, the excess of more than five times greater preoperative value as the index of perioperative MI, no patients in neither groups had perioperative MI. Pre and post operative echocardiographic study of EF showed no significant difference between the two groups nor in each group per se (table 1). 

**Table1:** Pre and postoperative echocardiographic data (ejection fraction and wall motion score index) in warfarin-aspirin and clopidogrel-aspirin groups

	**Warfarin – aspirin** **Mean±SD**	**Clopidogrel – aspirin** **Mean±SD**
Preoperative EF * Late postoperative EF	42.63±10.97 41.05±10.74	42.76±7.97 44.83±8.71
P-value	0.316	0.212
Preoperative WMSI ** Postoperative WMSI	1.88±0.47 1.83±0.6	2.01±0.47 1.79±0.46
P-value	0.477	0.001

* EF: Ejection Fraction,

** WMSI: Wall motion score index

As regards total heart WMSI to compare pre and 3 months postoperation, a significant difference was seen in clopidogrel – aspirin group (p- value less than 0.001) but not in warfarin – aspirin group (table 1). The same result has been obtained about local heart WMSI in territory of LAD and RCA. In other words, in regard to local heart WMSI to compare pre and 3 months post operation, there was a significant difference in clopidogrel – aspirin group but not in warfarin – aspirin group. 

## Discussion

There are a few studies in literature evaluating anticoagulation protocol in post CABG / CE patients, therefore, we decided to compare two anticoagulation protocol (“platelet “ inhibitor and “factor “ inhibitor) in these patients. CE is a known surgical technique required in some CABG patients with severe and diffuse atherosclerotic coronary involvement. 

The main concern regarding in this technique is the patency rate of grafting to such vessels postoperatively. As routine practice, it is recommended that such patients should be anticoagulated with warfarin for at least 3 months postoperatively which is expected time for complete neoendothelialization of endarterectomized vessels. Since morbidity and sometimes mortality of “warfarin toxicity “ necessitates closed monitoring of drug serum activity with serial INR checking, an alternative anticoagulant can be considered if it would have same effect without such problems. This study aimed to show that whether the anticoagulative effects of anti platelet agents such as clopidogrel, could be sufficient for the prevention of thrombosis of endarterectomized vessels during the time required for neoendothelialization. Principally, definitive evaluation of graft patency requires coronary catheterization directly, but generally it is not recommended if there is no clinical indication. Therefore in this study, we have employed indirect tools to evaluate graft patency. Postoperative ECG changes (new Q wave or new ST-T changes) in addition to increased serum CPK (MB) level (more than 5 times preoperative value) were considered as indicators of perioperative MI in early postoperative period. On the other hand, total or local WMSI in a special coronary artery territory was considered as such indicator in late (3 months) postoperative period. Most studies of CE in the literature have compared CABG / CE patients with CABG alone. Some studies have evaluated outcomes of CABG / CE patients. As mentioned earlier, there are a few studies evaluating post CABG / CE anti coagulation protocol. Thus, we cannot compare this study to similar others properly. 

Some studies evaluating post CABG / CE clinical course indicated that postoperative outcome in CABG patients with or without CE does not differ significantly (10). Hence, although patients requiring CE generally have higher cardiac risk profile and more severe coronary atherosclerotic involvement, CE is a preferred surgical option to obtain full myocardial revascularization. On the contrary, some other studies reported more mortality in patients with CABG / CE compared with CABG alone and consequently, it has been recommended that the addition of CE to CABG requires more caution (11). In our experience, CE was not accompanied with any graft thrombosis because there was no early nor late perioperative MI, so it seems that higher rate of morbidity and mortality of CABG / CE patients is related to more severe coronary artery atherosclerotic involvement of these patients due to higher cardiac risk profile and longer time coronary artery disease and CE per se is not the cause of increasing risk in such patients. In a study to consider late angiographic result of CABG / CE patients, it was shown that although adding warfarin to aspirin in postoperative anticoagulation therapy increases hemorrhagic risk, it is necessary to recommend it to maintain such patent graft, but this study did not compare warfarin to other drugs like clopidogrel (12). In another study, in the interim emphasis on lack of studies evaluating post CABG / CE anticoagulation protocol, two forms of management were carried out. In the first half of the study, patients were anticoagulated with warfarin – aspirin for 3 months post operation and then with aspirin alone. In the last half of the study, 3 months warfarin – aspirin was followed by clopidogrel – aspirin permanently. Since the design of the study was to evaluate early and late outcomes of CE in CABG / CE patients, it could not compare anti coagulation effects of two regimens warfarin – aspirin and clopidogrel – aspirin. This study concluded that because of disturbed endothelial integrity in endarterectomized vessels, aspirin alone could not maintain long-term such graft patency (13).

Yet there is not enough evidence to assume that endothelial manipulation in CE is somewhat similar to endothelial changes that occur in some other conditions such as during percutaneous coronary intervention (PCI) or during acute coronary syndromes. There are numerous reports that showed such senarios aspirin alone is not sufficient and adding some other agents is necessary (14, 15). As routine practice, it is recommended that heparin in acute coronary syndromes, and clopidogrel in PCI patients should be added to aspirin. In CE patients, warfarin is routinely used along with aspirin, but in this study, clopidogrel was considered to compare two protocols: warfarin – aspirin and clopidogrel – aspirin. Since re–catheterization of patients in post operative period is not routinely recommended, evaluation of graft patency was limited to indirect evidence such as ECG changes, CPK (MB) levels and WMSI alterations. The primary aim of this study was to compare two anticoagulation protocols and warfarin is used until 3 months post operation routinely, as a result we decided to limit the study in this period of time. But we suggest complementary and future studies with larger sample size and longer follow-up period beyond 3 months evaluating clopidogrel – aspirin compared with aspirin alone.

In CABG / CE patients, graft patency rate in early post operative period is similar in two anticoagulation protocols warfarin and clopidogrel. In late ( 3 months)postoperation, WMSI was significantly better in clopidogrel patients; so warfarin in spite of clopidogrel has serious hemorrhagic complications and requires close monitoring of serum drug activity with serial INR checking, it is suggested that clopidogrel – aspirin can be a preferred alternative anticoagulation therapy in CABG / CE patients. 
